# Glucocorticoid therapy for sepsis in the AI era: a survey on current and future approaches

**DOI:** 10.1016/j.csbj.2024.04.020

**Published:** 2024-04-12

**Authors:** Chenglong Liang, Shuo Pan, Wei Wu, Fanxuan Chen, Chengxi Zhang, Chen Zhou, Yifan Gao, Xiangyuan Ruan, Shichao Quan, Qi Zhao, Jingye Pan

**Affiliations:** a The First Affiliated Hospital of Wenzhou Medical University, Wenzhou 325000, China; b Wenzhou Medical University, Wenzhou 325000, China; c School of Nursing, Wenzhou Medical University, Wenzhou 325000, China; d School of Computer Science and Software Engineering, University of Science and Technology Liaoning, Anshan 114051, China; e School of Biomedical Engineering, School of Ophthalmology and Optometry, Eye Hospital, Wenzhou Medical University, Wenzhou 325000, China; f School of Materials Science and Engineering, Shandong Jianzhu University, Jinan 250101, China; g Department of Intensive Care Unit, The First Affiliated Hospital of Wenzhou Medical University, Wenzhou 325000, China; h Department of Big Data in Health Science, The First Affiliated Hospital of Wenzhou Medical University, Wenzhou 325000, China; i Key Laboratory of Intelligent Treatment and Life Support for Critical Diseases of Zhejiang Province, Wenzhou 325000, China; j Wenzhou Key Laboratory of Critical Care and Artificial Intelligence, Wenzhou 325000, China; k Zhejiang Engineering Research Center for Hospital Emergency and Process Digitization, Wenzhou 325000, China

**Keywords:** Sepsis, Glucocorticoid therapy, Artificial intelligence, Therapeutic strategies

## Abstract

Sepsis, a life-threatening medical condition, manifests as new or worsening organ failures due to a dysregulated host response to infection. Many patients with sepsis have manifested a hyperinflammatory phenotype leading to the identification of inflammatory modulation by corticosteroids as a key treatment modality. However, the optimal use of corticosteroids in sepsis treatment remains a contentious subject, necessitating a deeper understanding of their physiological and pharmacological effects. Our study conducts a comprehensive review of randomized controlled trials (RCTs) focusing on traditional corticosteroid treatment in sepsis, alongside an analysis of evolving clinical guidelines. Additionally, we explore the emerging role of artificial intelligence (AI) in medicine, particularly in diagnosing, prognosticating, and treating sepsis. AI's advanced data processing capabilities reveal new avenues for enhancing corticosteroid therapeutic strategies in sepsis. The integration of AI in sepsis treatment has the potential to address existing gaps in knowledge, especially in the application of corticosteroids. Our findings suggest that combining corticosteroid therapy with AI-driven insights could lead to more personalized and effective sepsis treatments. This approach holds promise for improving clinical outcomes and presents a significant advancement in the management of this complex and often fatal condition.

## Graphical abstract

**Figure:**
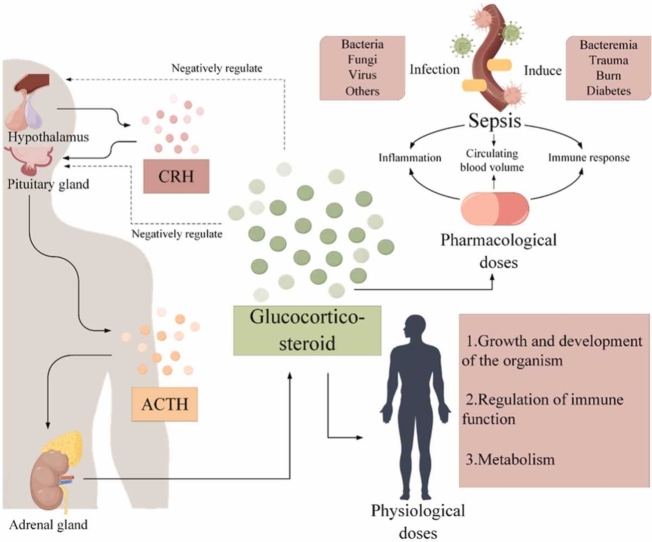

## 1 Introduction

### 1.1 Definition and epidemiology of sepsis

Sepsis, a disease that has undergone thousands of years of evolution in the history of medicine, is considered to be a typical disease of our era, and it is the main cause of disability and death from infection. It is characterized by a life-threatening organ dysfunction resulting from a dysregulated host response to infection [1]. The history of sepsis can be traced back at least to the time of Hippocrates [2]. Globally, sepsis-related fatalities are among the leading causes of death worldwide, responsible for almost one-fifth of all deaths and a predominant cause of fatalities in hospital intensive care units. As of 2020, Lancet data revealed an annual global incidence of 48.9 million sepsis cases, with approximately 11 million deaths, constituting 19.7 % of global deaths [3]. Over the span of 1990 to 2017, there has been a noteworthy reduction in the incidence and mortality of sepsis by 37.0 % and 52.8 %, respectively. Geographical disparities in the burden of sepsis are evident, with sub-Saharan Africa, Oceania, South Asia, and East and Southeast Asia experiencing the most pronounced impact. Economic implications are also significant, with an estimated annual healthcare expenditure of US$24 billion attributable to sepsis [4]. The underlying pathophysiology of sepsis is multifaceted, involving intricate interactions among the host's immune system, coagulation pathways, endothelial functions, and microbial components [5]. The mortality associated with sepsis has seen a gradual decline over recent decades owing to timely interventions such as antibiotics, fluid resuscitation, and multi-organ support therapies. Nevertheless, the mortality rate remains considerable, and the therapeutic landscape of sepsis continues to present opportunities for enhancement [6].

### 1.2 Role and potential of glucocorticoids in the treatment of sepsis

Since the 1990s, glucocorticoids have emerged as a distinctive therapeutic agent in the sepsis treatment landscape [7]. In recent years, numerous randomized controlled trials have assessed the efficacy of low-dose hydrocortisone for septic patients, demonstrating its potential to curtail the duration of shock. However, findings related to mortality impact remain inconsistent, and the definitive role of glucocorticoids in sepsis care continues to be a subject of controversy and non-standardization [8], [9]. Absence of unified criteria for the administration of glucocorticoids further accentuates this ambiguity. Hence, the precise application of glucocorticosteroids in sepsis requires verification through robust and comprehensive research endeavors.

### 1.3 Overview and significance of the development and application of artificial intelligence in medicine

The growing utilization of AI within the medical domain marks a significant advancement in healthcare delivery. By harnessing machine learning algorithms to parse medical data, AI augments both diagnostic and therapeutic processes. Its applications primarily span clinical decision support [10] and medical image analysis [11].

Clinical decision support systems equip healthcare providers with swift access to pertinent patient information, thereby facilitating informed decisions about treatment modalities, medication choices, mental health interventions, and more. These tools can provide physicians with personalized treatment recommendations by integrating large amounts of medical data, medical record information, lab results, and others. Concurrently, in the field of medical imaging, AI algorithms excel in scrutinizing CT scans, X-rays, MRI images, and so on, to identify potential lesions or findings that may elude radiologists [12]. When implemented judiciously, AI-driven decision support mechanisms enhance patient safety through error minimization, patient stratification, and proficient medication management [13].

Despite these advancements, AI's engagement in sepsis glucocorticoid therapy is still in its early stages. This scarcity may be attributed to sepsis's intricate progression and nebulous disease definition. As a serious infectious disease with multifaceted pathogenesis, sepsis's diagnosis and treatment pose substantial challenges. Personalized and adaptive strategies are essential in managing sepsis, necessitating consideration of patient-specific conditions and variances, along with timely adjustments of treatment protocols to improve survival and prognosis.

The advent of AI within this context may herald innovative breakthroughs in sepsis glucocorticoid therapy. By analytically probing extensive clinical data and research findings, AI can render precise decision support, thereby optimizing sepsis treatment plans. The use of AI with glucocorticoid therapy promises to augment therapeutic outcomes, utilizing personalized, data-driven insights to enrich patient care.

### 1.4 Outline and objectives of this review

In the present review, we embark on a systematic examination of the prospective evolution of AI in sepsis glucocorticoid therapy. Through an integrative synthesis and analytical assessment of extant research findings and clinical experiences, we endeavor to elucidate AI's potential in enhancing sepsis patient outcomes, bolstering survival rates, and refining prognosis accuracy.

This comprehensive review offers a nuanced portrayal of the existing state of the art, illuminating the complexities, potentials, and impediments of AI application within the ambit of sepsis glucocorticoid therapy. Moreover, it outlines future research directives, probing the prospects, challenges, and viable strategies to further the integration of AI within this domain.

Our primary intent lies in furnishing clinicians and researchers with an instructive reference, fostering the progression towards personalized and precise sepsis glucocorticoid therapy. We advocate for a concerted push towards AI-based methodologies, with the aspiration of rendering more sophisticated and tailored guidance for the therapeutic management of sepsis.

## 2 Current status of glucocorticoids in sepsis treatment

### 2.1 Physiologic and pharmacologic effects of glucocorticoids

**Fig. 1:**
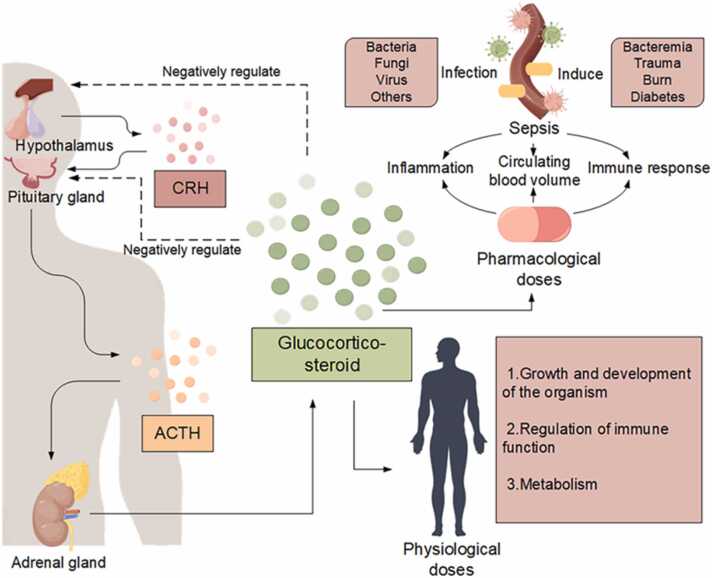
Glucocorticoids are adrenocorticotropic hormones that regulate substance synthesis and metabolism via the hypothalamic-pituitary-adrenal (HPA) axis, as well as anti-inflammatory effects. It has an important role in sepsis. In patients with sepsis, the immune system is activated to eliminate the source of infection. However, if not properly balanced, pathogen clearance is associated with excessive inflammation leading to organ failure. Sepsis also leads to dysregulation of the immune response and consequent metabolic changes, such as changes in blood glucose. Severe sepsis can also lead to circulatory instability and signs of shock. Glucocorticoids are induced during infection to maintain homeostasis and to withstand the life-threatening effects of sepsis on the host.

Glucocorticoids constitute a class of steroid hormones that are either secreted by the zona fasciculata in the intermediate layer of adrenal cortex or synthesized chemically. These hormones exert crucial regulatory influence over the biosynthesis and metabolism of carbohydrates, lipids, and proteins through the coordinated function of the hypothalamic-pituitary-adrenal (HPA) axis, alongside demonstrated anti-inflammatory properties [14]. As integral components within the human physiological system, the regulatory activities of glucocorticoids can be modulated by diverse elements, including serotonin, gamma-aminobutyric acid (GABA), and pro-inflammatory cytokines, when present in physiological concentrations. Their multifaceted roles span the regulation of developmental processes, growth dynamics, metabolic homeostasis, and immune function (Fig. 1) [15]. Pharmacologically, glucocorticoids are principally harnessed for their potent anti-inflammatory, immunosuppressive, and anti-shock properties. This is evidenced in their broad application across therapeutic contexts, targeting inflammatory ailments, where they act via several effector molecules, contribute to lysosomal membrane stabilization, inhibit the release of pro-inflammatory mediators, and augment vasoconstriction to remedy vascular paralysis [16], [17], [18], [19]. The widespread use of glucocorticoids underscores the significance of glucocorticoids in the contemporary treatment paradigm for inflammatory diseases.

### 2.2 Mechanisms of action, effects and side effects of glucocorticoids in the treatment of sepsis

In the modern medical landscape, the role of glucocorticoids in modulating the inflammatory response, especially in patients with sepsis, has become a focal point of research. Glucocorticoids exert their effects by binding to the intracellular glucocorticoid receptor (GR), a nuclear transcription factor that plays a regulatory role in the transcription of nearly 20 % of the protein-coding genes within human leukocytes [20]. Emerging data from initial observational clinical investigations, along with results derived from preclinical sepsis models, underline the significant phenomenon of glucocorticoid resistance (GCR) during sepsis. This resistance may be ascribed to a decline in GR expression or responsiveness [21].

The therapeutic employment of glucocorticoids in sepsis has engendered intense debate and divergent viewpoints. A comprehensive meta-analysis conducted in 2019 illustrated that the administration of long-term low-dose corticosteroids appeared to diminish 28-day mortality rates, ICU (intensive care unit) mortality, and the length of ICU stays [22]. Concurrently, research from 2019 also revealed potential side effects associated with glucocorticoid therapy. Specifically, it elucidated an increased risk of muscle weakness (21 % heightened risk in the hormone-treated group versus controls) and hypernatremia (49 % escalated risk in the hormone-treated group in comparison to controls), alongside a possible augmentation in the risk of hyperglycemia (12 % elevated risk in the hormone-treated group relative to controls), simply put, the group treated with glucocorticoids had an increased risk of myasthenia gravis, hypernatremia, hyperglycemia [23]. These findings collectively underscore the necessity for a nuanced and judicious approach to glucocorticoid therapy in sepsis, integrating both its potential benefits and associated risks.

### 2.3 Research developments in glucocorticoid treatment of sepsis

The role of corticosteroids in treating septic shock has undergone extensive research and debate, evidenced by diverse findings from numerous studies over the years. The relevant RCT literature mentioned is presented in Table 1.

**Table 1:** RCT studies of corticosteroids in sepsis (by year).

Title	Journal abbreviation	First author	Publication year	Corticosteroid regimen	Conclusion	Reference
Steroids in the treatment of clinical septic shock	Ann Surg	Schumer	1976	3 mg/kg dexamethasone or 30 mg/kg methylprednisolone	High-dose corticosteroids significantly reduced mortality.	[24]
The effects of high-dose corticosteroids in patients with septic shock. A prospective, controlled study	N Engl J Med	Sprung	1984	Methylprednisolone 30 mg/kg for 10-15 min; dexamethasone, 6 mg/kg for 10-15 min	Corticosteroids do not improve survival rates in severe septic shock, but they may be beneficial in the early stages or for certain subgroups.	[25]
Effect of high-dose glucocorticoid therapy on mortality in patients with clinical signs of systemic sepsis	N Engl J Med	Veterans Administration Systemic Sepsis Cooperative Study Group	1987	Methylprednisolone, 30 mg/kg over 15 min) and 9-hour IV infusion (5 mg/kg/h)	Early high-dose steroids do not reduce mortality in systemic sepsis and should not be used as adjunct therapy.	[27]
A controlled clinical trial of high-dose methylprednisolone in the treatment of severe sepsis and septic shock	N Engl J Med	Bone	1987	Methylprednisolone, 30 mg/kg/6 h for 4 times.	High-dose steroids aren't beneficial for severe sepsis and shock.	[26]
Detrimental effects of high-dose methylprednisolone sodium succinate on serum concentrations of hepatic and renal function indicators in severe sepsis and septic shock. The Methylprednisolone Severe Sepsis Study Group	Crit Care Med	Slotman	1993	Methylprednisolone, 30 mg/kg/6 h for 4 times.	High-dose methylprednisone heightens risk of increased blood urea and bilirubin in sepsis; its adverse effects should guide treatment plans.	[28]
Reversal of late septic shock with supraphysiologic doses of hydrocortisone	Crit Care Med	Bollaert	1998	Hydrocortisone, 100 mg/d for 5d	Moderate hydrocortisone improves hemodynamics and survival in septic shock, unrelated to adrenocortical insufficiency.	[29]
Stress doses of hydrocortisone reverse hyperdynamic septic shock: a prospective, randomized, double-blind, single-center study	Crit Care Med	Briegel	1999	Hydrocortisone, 100 mg administered over 30 min and maintained thereafter at a continuous infusion dose of 0.18 mg/kg/h	Stress-dose hydrocortisone reduces vasopressor time in septic shock, hinting early organ dysfunction improvement; no mortality difference observed.	[33]
Effect of treatment with low doses of hydrocortisone and fludrocortisone on mortality in patients with septic shock	JAMA	Annane	2002	Hydrocortisone, 50 mg/6 h for seven days	Low-dose hydrocortisone and fludrocortisone for 7 days lower death risk in septic shock without more adverse events.	[31]
Physiological-dose steroid therapy in sepsis [ISRCTN36253388]	Crit Care	Yildiz	2002	Prednisolone, 5 mg/12 h for 10 days	Physiologic steroids hint at decreased sepsis mortality; adrenocortical insufficiency in septic progression is less frequent than expected.	[41]
Immunologic and hemodynamic effects of "low-dose" hydrocortisone in septic shock: a double-blind, randomized, placebo-controlled, crossover study	Am J Respir Crit Care Med	Keh	2003	First 100 mg hydrocortisone as loading dose, then 10 mg/h until day 3	Avoid rebound by gradually reducing low-dose hydrocortisone.	[46]
Low-dose hydrocortisone improves shock reversal and reduces cytokine levels in early hyperdynamic septic shock	Crit Care Med	Oppert	2005	Hydrocortisone, 50 mg post-injection continuous drip, reduced after reversal of shock	Low-dose hydrocortisone accelerates early septic shock reversal and reduces pro-inflammatory cytokines, implying the blood flow dynamics and immunomodulatory effects of steroid treatment.	[30]
Adrenal insufficiency in patients with cirrhosis and septic shock: Effect of treatment with hydrocortisone on survival	Hepatology	Fernández	2006	Hydrocortisone, 50 mg/6 h.	Advanced cirrhosis patients often have adrenocortical insufficiency; hydrocortisone use boosts shock resolution and survival.	[32]
Effect of low doses of corticosteroids in septic shock patients with or without early acute respiratory distress syndrome	Crit Care Med	Annane	2006	Hydrocortisone 50 mg/6 h for 7 days	Low-dose corticosteroids for 7 days benefit early ARDS nonresponders in septic shock, not responders or those without ARDS.	[42]
Effect of mode of hydrocortisone administration on glycemic control in patients with septic shock: a prospective randomized trial	Crit Care	Loisa	2007	Hydrocortisone, 50 mg/6 h and 200 mg/d continuous infusion, observed for 5 days	Hydrocortisone infusion in septic shock patients aids in strict glycemic control and eases nursing workload.	[39]
Impact of bolus application of low-dose hydrocortisone on glycemic control in septic shock patients	Intensive Care Med	Weber-Carstens	2007	Hydrocortisone, 50 mg/6 h (1 dose) and 200 mg/d continuous infusion	Pushing hydrocortisone elevates blood glucose variably in septic shock patients. Continuous infusion aids consistent glycemic control.	[40]
Hydrocortisone therapy for patients with septic shock	N Engl J Med	Sprung	2008	Hydrocortisone, 50 mg/6 h for 5 days, followed by continuous tapering	Hydrocortisone didn't boost survival or shock reversal but sped up shock reversal in those already reversing.	[34]
Low-dose hydrocortisone in patients with cirrhosis and septic shock: a randomized controlled trial	CMAJ	Arabi	2010	Hydrocortisone, 50 mg/6 h until blood flow stabilizes and then decreases (within 8d)	In cirrhotic patients with septic shock, hydrocortisone improves hemodynamics but doesn't lower mortality and increases adverse effects.	[35]
Low-dose hydrocortisone treatment for patients with septic shock: a pilot study comparing 3days with 7days	Respirology	Huh	2011	Hydrocortisone, 50 mg/6 h for 3d or 7d	Low-dose hydrocortisone for 28 or 3 days showed no difference in 7-day mortality in septic shock patients.	[45]
Time course of organ failure in patients with septic shock treated with hydrocortisone: results of the Corticus study	Intensive Care Med	Moreno	2011	Hydrocortisone, 50 mg/6 h intravenously, dose tapered to 11 days after five days	Hydroxycortisone improved SOFA scores and cardiovascular function but didn't reduce mortality.	[36]
Effect of Hydrocortisone on Development of Shock Among Patients With Severe Sepsis: The HYPRESS Randomized Clinical Trial	JAMA	Keh	2016	Hydrocortisone, continuous infusion of 200 mg/d, dose tapered to 11 days after five days	Hydrocortisone didn't reduce septic shock risk within 14 days in adults with severe sepsis; its use is not supported.	[44]
Hydrocortisone plus Fludrocortisone for Adults with Septic Shock	N Engl J Med	Annane	2018	Hydrocortisone (IV push 50 mg/6 h non-decreasing for seven days) plus fludrocortisone (tablets, 50 mcg qm given by nasogastric tube)	In septic shock patients, the 90-day mortality was lower with hydrocortisone plus fludrocortisone than placebo.	[101]
Adjunctive Glucocorticoid Therapy in Patients with Septic Shock	N Engl J Med	Venkatesh	2018	Continuous intravenous hydrocortisone infusion (200 mg/d for 7 days or death, discharge)	In ventilated septic shock patients, 90-day mortality with hydrocortisone wasn't lower than placebo.	[37]
Differential Response to Steroids. From the VANISH Randomized Trial	Am J Respir Crit Care Med	Antcliffe	2019	One group received an initial 100 mg of hydrocortisone as a loading dose, followed by 10 mg every hour until day 3 (n = 20), and the other group received placebo (n = 20) and then switched to the opposite drug by day 6	Septic shock transcriptomic profiles correlate with corticosteroid response. The immunocompetitive SRS2 phenotype had higher mortality with corticosteroids than placebo.	[49]
Hydrocortisone Compared with Placebo in Patients with Septic Shock Satisfying the Sepsis-3 Diagnostic Criteria and APROCCHSS Study Inclusion Criteria: A Post Hoc Analysis of the ADRENAL Trial	Anesthesiology	Venkatesh	2019	Hydrocortisone group (200 mg/d continuous infusion for 7d)	In Sepsis-3 or APROCCHSS subjects, 90-day mortality with hydrocortisone wasn't lower than placebo.	[38]
Effect of Ascorbic Acid, Corticosteroids, and Thiamine on Organ Injury in Septic Shock: The ACTS Randomized Clinical Trial	JAMA	Moskowitz	2020	Combination, hydrocortisone, 50 mg/6 h for 96 h or until discharge or death	In septic shock patients, ascorbic acid-corticosteroid-thiamine combo didn't significantly lower SOFA scores versus placebo, not supporting its routine use.	[47]
Effect of Vitamin C, Thiamine, and Hydrocortisone on Ventilator- and Vasopressor-Free Days in Patients With Sepsis: The VICTAS Randomized Clinical Trial	JAMA	Sevransky	2021	Combination, hydrocortisone, 50 mg/6 h for 96 h or until discharge or death	In critical sepsis patients, vitamin C-thiamine-hydrocortisone didn't notably increase ventilator/vasopressor days versus placebo; trial ended early, possibly missing key differences.	[48]
External Corroboration That Corticosteroids May Be Harmful to Septic Shock Endotype A Patients	Crit Care Med	Wong	2021	Hydrocortisone 200 mg/d in 4 injections for 5 days	Corticosteroid exposure may raise mortality in type A septic shock patients; consistent with prior findings.	[50]
The relationship between adrenocortical candidate gene expression and clinical response to hydrocortisone in patients with septic shock	Intensive Care Med	Cohen	2021	Hydrocortisone (IV 50 mg/6 h for 7 days or until discharge or death.	Gene expression doesn't correlate with septic shock mortality. GLCCI1-high patients on hydrocortisone had faster shock remission, while BHSD1-high ones had slower.	[51]

In 1976, Schumer et al. [24] conducted a two-part study, both prospective and retrospective, to assess the safety and efficacy of corticosteroids in septic shock treatment. They found a significantly lower mortality rate in patients treated with high doses of corticosteroids (either 3 mg/kg dexamethasone or 30 mg/kg methylprednisolone). However, no significant mortality rate difference was observed between the two corticosteroids. Following up in 1984, Sprung et al. [25] in a smaller-scale prospective study, observed that high-dose corticosteroid-treated patients were more likely to experience early shock reversal during hospitalization compared to those untreated. They concluded that corticosteroids are potentially beneficial in the early stages and in specific patient subgroups, and that high-dose glucocorticoids may be effective in the treatment of sepsis and septic shock.

In 1987, Bone et al. [26] carried out a double-blind randomized controlled trial with 382 patients, revealing a higher mortality rate (34 %) in the group treated with high-dose glucocorticoids compared to the placebo group (25 %). The study also found an increased risk of death from secondary infections due to high-dose glucocorticoid use. In the same year, the Veterans Administration Systemic Sepsis Cooperative Study Group [27] conducted a similar trial with 223 sepsis patients. Their findings showed negligible differences in 14-day mortality rates between the steroid (21 %) and placebo (22 %) groups, along with a higher rate of secondary infections in the steroid group. Furthermore, a 1993 study by Slotman et al. [28], involving 382 patients across 19 research centers, found that high-dose methylprednisolone led to significant increases in blood urea nitrogen and bilirubin levels in patients with severe sepsis. These studies collectively demonstrate the associated adverse effects of methylprednisolone in critically ill patients, questioning the risk-benefit relationship of high-dose corticosteroids in the treatment of sepsis and septic shock.

The effectiveness of corticosteroids in septic shock treatment has been a contentious topic, including subsequent published trials with varying findings regarding the use of corticosteroids. In 1998, Bollaert et al. [29] reported a significant shock reversal and survival improvement in septic patients treated with hydrocortisone. At 7 days, there was a 47 % difference in shock reversal between the two groups (95 % confidence interval 17 % to 77 %; p = 0.007). Oppert et al. [30] in 2005 and Annane et al. [31] in 2002, as well as Fernández et al. [32] in 2006, found similar benefits, with no increase in adverse events. Contrastingly, Briegel et al. [33] (1999) observed no impact of hydrocortisone on shock reversal or mortality, though it might shorten vasopressor use. Sprung et al. [34] (2008) noted that hydrocortisone accelerated shock reversal without affecting survival. Arabi et al. [35] (2010) found hydrocortisone improved hemodynamics, patients in the hydrocortisone group had a higher rates of shock reversal (relative risk [RR] 1.58, 95 % confidence interval [CI] 0.98–2.55, p = 0.05). But not mortality in cirrhotic patients, with increased shock recurrence (RR 2.58, 95 % CI 1.04–6.45, p = 0.03) and gastrointestinal bleeding (RR 3.00, 95 % CI 1.08–8.36, p = 0.02). Moreno et al. [36] (2011) also reported no mortality reduction, although hydrocortisone sped up organ function recovery. Recent studies by Venkatesh [37], [38] in 2018 and 2019 showed that continuous hydrocortisone infusion led to quicker shock remission and fewer blood transfusions, though not affecting mortality. Patients in the experimental group also had shorter hospital stays and ventilator use.

The guidelines for corticosteroid administration in septic shock remain unclear, especially regarding the mode and regimen. Preliminary evidence suggests intravenous continuous infusion might be superior to push administration, as highlighted by Loisa [39] and Weber-Carstens [40] in 2007. Their studies showed continuous infusion maintained tighter glycemic control, avoiding the blood glucose spikes seen with intermittent push administration.

Regarding dosing regimens, there's no consensus on an optimal approach. Large doses have been largely abandoned in favor of smaller ones [24], [25], [26], [27], [28], but the effectiveness of these reduced doses is still debated. Yildiz et al. [41] in 2002 reported a declining mortality trend with physiological doses, though inconclusively. Annane et al. [42], [43], in studies conducted in 2006 and 2018, observed improved outcomes with low-dose hydrocortisone and fludrocortisone in certain patients.

Despite these findings, Sprung [34], Arabi [35], and Moreno [36] did not note mortality reductions with low-dose regimens. Keh et al. [44] in 2016 found no risk reduction within 14 days, and the optimal dosing duration remains unclear. Huh et al. [45] in 2011 also found no mortality difference between 3-day and 7-day low-dose treatments. While both abrupt and gradual corticosteroid cessation have been studied, there's no clear consensus on the best approach, although gradual tapering is often suggested [30], [34], [35], [36], [44]. Keh et al. [46], in their 2003 crossover study, reported a rebound effect post abrupt corticosteroid discontinuation.

Recent studies have also explored treatment combinations which include corticosteroids. Moskowitz et al. [47] in 2020 found that combining ascorbic acid, corticosteroids, and thiamine did not significantly change sequential organ failure assessment (SOFA scores) compared to placebo. Similarly, Sevransky et al. [48] in 2021 further validated that for severe sepsis patients, the trio of vitamin C, thiamine, and hydrocortisone didn't notably reduce the duration of ventilator and vasopressin utilization over a span of 30 days when compared with a placebo.

Recent findings highlight the heterogeneous corticosteroid response across sepsis subtypes. In a 2019 double-blind RCT examining septic shock patient response to norepinephrine or vasopressin alongside hydrocortisone or placebo, Antcliffe et al. [49] discovered a correlation between the transcriptome at septic shock onset and corticosteroid responsiveness. Notably, immunocompetent patients with SRS2 endotype displayed elevated mortality upon corticosteroid treatment compared to placebo. In a subsequent 2021 secondary analysis, Wong et al. [50] postulated potential detriment from hydrocortisone therapy in septic shock type A patients, based on public transcriptomic datasets. Concurrently, Cohen et al. [51] explored the relationship between adrenocortical-related gene expression and response to corticosteroid administration in septic shock. Their data indicated that hydrocortisone expedited shock remission in patients with heightened GLCCI1 expression but delayed it in those with increased BHSD1 expression. Such genetic expression variances might underpin the diverse responses to corticosteroid therapy observed in septic shock.

### 2.4 Guidelines and challenges in glucocorticoid treatment of septicemia

Since the introduction of the 2004 Surviving Sepsis Campaign guidelines for the management of severe sepsis and septic shock [52], the role of glucocorticoids in sepsis has become a contentious issue. A detailed examination of successive guidelines elucidates a continually evolving landscape, indicative of a field undergoing frequent reevaluations and refinements.

The 2004 guidelines [52] stated that intravenous corticosteroids were recommended for patients who, despite adequate fluid replacement, require vasopressor therapy to maintain adequate blood pressure during septic shock (200–300 mg of hydrocortisone per day in 3 or 4 divided doses or continuous infusion) and did not support the use of hydrocortisone in doses greater than 300 mg per day in the treatment of severe sepsis or septic shock. However, in a 2008 update of the guideline [53] it was noted that the ACTH stimulation test should not be used to identify the subset of adults (with relative adrenal insufficiency) who should receive hydrocortisone. Thus, there was no need to use dexamethasone until the ACTH test was performed. Immediately following the 2012 and 2016 guidelines [54], [55], it is considered that intravenous hydrocortisone is not recommended for the treatment of adult patients with infectious shock if appropriate fluid resuscitation and vasopressor therapy can restore hemodynamic stability. If recovery is not possible, intravenous hydrocortisone at 200 mg per day alone is recommended. This is a relatively more precise dose compared to the previous version of the guidelines. In the latest 2021 guidelines [56] mentions that intravenous corticosteroids are recommended for patients with shock who have an ongoing requirement for vasopressor therapy (200 mg of intravenous hydrocortisone per day, which can be given intravenously at 50 mg every 6 h or as a continuous infusion. It is recommended that it be initiated at least 4 h after the administration of norepinephrine or an epinephrine dose of ≥0.25 mg/kg/min). Although this recommendation is a weak recommendation with moderate quality evidence in the guideline. It is worth noting that this is the first time that the SSC guidelines have proposed a minimum dose of vasopressor and time of therapy which prompts the initiation of corticosteroids.

A conspicuous absence across these guidelines is high-quality evidence delineating the precise dosage and administration of glucocorticoids. Table 2 provides a comprehensive summary of the recommendations in conjunction with their respective evidence levels (C and above).

**Table 2:** Corticosteroid recommendations in various versions of the guidelines.

Guideline	Corticosteroid recommendations	Grade of evidence	Reference
2004	Intravenous corticosteroids (hydrocortisone 200–300 mg/day, for 7 days in 3 or 4 divided doses or by continuous infusion) are recommended in patients with septic shock who, despite adequate fluid replacement, require vasopressor therapy to maintain adequate blood pressure	Grade C	[52]
	Doses of corticosteroids higher than > 300 mg hydrocortisone daily should not be used in severe sepsis or septic shock for the purpose of treating septic shock	Grade A	
2008	We suggest intravenous hydrocortisone be given only to adult septic shock patients after blood pressure is identified to be poorly responsive to fluid resuscitation and vasopressor therapy	Grade 2 C	[53]
	We suggest the ACTH stimulation test not be used to identify the subset of adults with septic shock who should receive hydrocortisone	Grade 2B	
	We suggest that patients with septic shock should not receive dexamethasone if hydrocortisone is available	Grade 2B	
	We suggest the daily addition of oral fludrocortisone (50 μg) if hydrocortisone is not available and the steroid that is substituted has no significant mineralocorticoid activity. Fludrocortisone is considered optional if hydrocortisone is used	Grade 2 C	
	We recommend doses of corticosteroids comparable to > 300 mg hydrocortisone daily not be used in severe sepsis or septic shock for the purpose of treating septic shock	Grade 1 A	
2012	We suggest not using intravenous hydrocortisone as a treatment of adult septic shock patients if adequate fluid resuscitation and vasopressor therapy are able to restore hemodynamic stability (see goals for Initial Resuscitation). If this is not achievable, we suggest intravenous hydrocortisone alone at a dose of 200 mg per day	Grade 2 C	[54]
	We suggest not using the ACTH stimulation test to identify the subset of adults with septic shock who should receive hydrocortisone	Grade 2B	
2016	We suggest against using IV hydrocortisone to treat septic shock patients if adequate fluid resuscitation and vasopressor therapy are able to restore hemodynamic stability. If this is not achievable, we suggest IV hydrocortisone at a dose of 200 mg per day	weak recommendation, low quality of evidence	[55]
2021	For adults with septic shock and an ongoing requirement for vasopressor therapy we suggest using IV corticosteroids	Weak recommendation; moderate quality of evidence	[56]

Current investigations denote promising clinical efficacy for small-dose glucocorticoids in treating septic shock such as reducing the duration of shock, mechanical ventilation and ICU stay[9], [57]. Nonetheless, a cloud of controversy persists around the use of glucocorticosteroid in sepsis patients. Acknowledging the multifaceted pathogenesis of sepsis, its swift progression, often cryptic etiology, and substantial patient variability presents formidable obstacles to the medical community in devising a standardized treatment protocol for glucocorticoid utilization. These complexities highlight the challenges inherent in the diagnosis and treatment of a condition that resists simple categorization, further underscoring the evolving and debated role of glucocorticoids in sepsis.

## 3 Future applications of glucocorticoids in the treatment of sepsis

### 3.1 Current development and application of AI in the medical field

The term 'Artificial Intelligence' emerged in 1956, conceived by John McCarthy during a seminal conference dedicated to the subject [58]. Over subsequent decades, AI has undergone transformative evolution, marking a transition from nascent concept to a technological juggernaut. With machine learning and deep learning at its core, AI has ventured into domains previously uncharted, most notably in the field of personalized medicine [59].

In an era inundated with data, where the sheer volume of information often eclipses human cognitive processing capabilities, AI has emerged as a pivotal instrument. It not only complements human cognition but plays an indispensable role in shaping personalized healthcare delivery systems [60]. AI is currently showing a relatively high level of proficiency in imaging and signal detection tasks, and is considered to be one of the most sophisticated tools in the field [61]. The advent of the 5 G era has brought about an amplification of AI's capabilities, particularly in the arenas of image recognition [62]. drug design and discovery [63], disease diagnosis [64], [65], neurology [66], and numerous other interdisciplinary fields. It has led to a profound synergy between various scientific disciplines, fostering integration and development in ways previously unimagined.

AI now commands a role that transcends mere assistance. It is pivotal in enhancing diagnostic accuracy, minimizing misdiagnoses, augmenting efficiency, equilibrating the imbalance between resource supply and demand, providing early warning for disease risk, and invigorating drug development. Its influence permeates the medical field, setting the stage for a future where AI's integration into healthcare is not merely a possibility but an expectation.

### 3.2 Application of AI in the field of sepsis

According to the survey, the applications of AI in the field of sepsis can be broadly categorized into the following major applications: early detection of sepsis, prediction of sepsis complications, prediction of sepsis mortality, diagnosis and risk stratification by classifying subtypes of sepsis, and screening of sepsis-related biomarkers.

#### 3.2.1 Early detection of sepsis

Kam et al. [67] (2017) demonstrated the efficacy of deep learning methodologies by developing deep neural networks using the MIMIC-II database, which outperformed traditional models AUCs (area under ROC curve) of 0.915 and 0.929 for DFN (SepDFN100) and LSTM (SepLSTM) models, respectively. Aguirre et al. [68] (2022) further validated this by assessing seven models using routine blood tests in suspected sepsis cases, where all models exceeded an AUC of 0.9, notably the multi-layer perceptron model with 0.95. Their findings affirm that machine learning and AI models are superior in early sepsis prediction, though emphasizing the need for external validation for broader applicability.

#### 3.2.2 Prediction of sepsis complications

In predicting sepsis complications, Javan et al. [69] (2019) showcased a machine learning approach for forecasting cardiac arrest in sepsis patients, utilizing methods like logistic regression, SVM (support vector machine), decision trees, random forests, and XGBoost with MIMIC-III data. By innovatively using stacking algorithms and multivariate dataset, they achieved over 70 % accuracy and sensitivity for predicting cardiac arrest up to six hours earlier. The findings emphasize the significance of time-series dynamics of vital signs in predicting cardiac arrest in sepsis patients. Zhang et al. [70] (2022) conducted a retrospective cohort investigation, employing XGBoost to anticipate acute kidney injury (AKI) in septic shock patients, yielding an AUC of 0.821, demonstrating its efficacy over traditional scores like SOFA and APACHE II (acute physiology and chronic health evaluation). Concurrently, Yue et al. [71] explored various models including logistic regression, k-nearest neighbor, SVM, decision tree, random forest, XGBoost, and neural networks for AKI prediction in sepsis, and obtained area under the receiver operating characteristic curves of 0.7365, 0.6637, 0.7353, 0.7492, 0.7787, 0.7547, 0.821, 0.6457 and 0.7015. The AUC of KNN was < 0.7 but higher than SOFA and < Saps 2. The AUC of XGBoost was 0.821 These results, aligning closely with those of Zhang et al. [70], reinforce the conclusion that machine learning models hold promise as trustworthy instruments for AKI prediction in sepsis patients.

#### 3.2.3 Diagnosis and risk stratification

In 2022, Qi et al. [72] applied five advanced machine learning algorithms, including Lasso regularization, Bayesian regression, decision tree, random forest, and XGBoost, to predict the in-hospital mortality rate of diabetic patients with septic shock, on over 7000 patients from three databases (MIMIC-IV, eICU-CRD, dtChina). These models underwent internal and external validations, highlighting the random forest's robustness in training and validation. Concurrently, Zhou et al. [73] analyzed data from 1936 patients (including 1692 sepsis samples and 244 normal samples) using weighted gene co-expression network analysis (WGCNA) to identify candidate genes related to sepsis and distinguishes two sepsis subtypes, Adaptive and Inflammatory, through K-means clustering analysis in a training set. The study found that the Adaptive subtype is associated with high levels of T cell and NK cell infiltration and better clinical outcomes, while the Inflammatory subtype correlates with high macrophage infiltration and poor prognosis. A risk score system was established and validated to independently predict sepsis patient outcomes. The nomogram analysis demonstrated superior ability to identify high-risk patients compared to age, gender, and risk score. These research findings reveal the potential of transcriptome analysis in personalized treatment and prognostication of sepsis.

#### 3.2.4 Screening of sepsis-related biomarkers

**Fig. 2:**
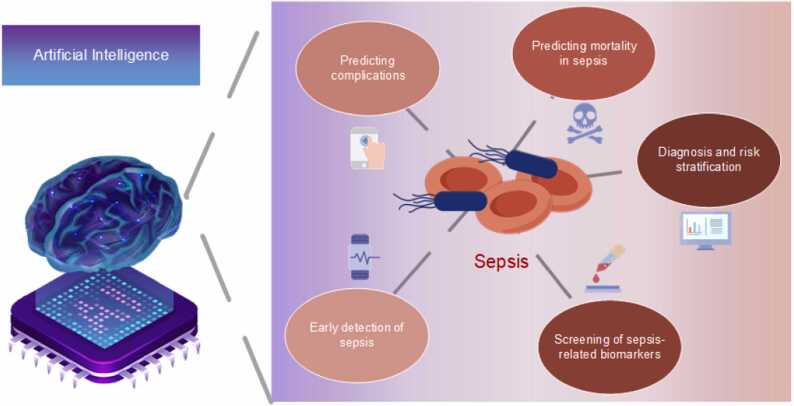
In our research on AI in sepsis, we delineate five main aspects of sepsis. At the same time, we elucidate some of the most widely used AI models in this research area.

Complementing this research, Chen et al. [74] conducted the role of costimulatory molecule genes (CMGs) in sepsis, utilizing machine learning algorithms to analyze data from GEO and Array Express datasets. They identified six key CMGs and developed a CMG classifier, which showed strong diagnostic and prognostic capabilities for sepsis, surpassing traditional markers like PCT and CRP in some instances. The classifier also helped in discerning the immune microenvironment in sepsis, correlating with different immune cell infiltrations and molecular pathways. This research underlines the potential of using CMGs for improved sepsis diagnosis, prognosis, and understanding of its immune landscape, potentially guiding individualized treatments. In summation, the evolving landscape of AI has catalyzed a transformative shift in the field of sepsis research. AI modeling is being progressively harnessed across multifaceted aspects of sepsis, encompassing the early detection of sepsis, identification of sepsis-related complications, mortality prediction, and more (Fig. 2). Intriguingly, the majority of these contemporary studies underscore an enhanced performance of AI-driven models compared to conventional methodologies. However, it is pertinent to note that most of the existing literature pivots on retrospective analyses conducted on public databases. Therefore, while the initial findings are promising, they warrant further exploration and validation through more in-depth, prospective research to fortify the evidence base. Such rigorous inquiry will be instrumental in translating these pioneering AI technologies into meaningful clinical interventions that could reshape the management and prognosis of sepsis.

### 3.3 AI-enhanced glucocorticoid treatment for sepsis

Historically, the individual administration of glucocorticoids has rested heavily on the clinician's own insights and personal experience. This subjective approach has resulted in certain challenges regarding the quantitative migration of this experience, culminating in certain discrepancies in dosing across different medical practitioners. The absence of patient-specific regression observables after glucocorticoid administration has further accentuated this issue. Contrastingly, the era of AI offers a remarkable advantage: the ability to model complex datasets, discovering both linear and non-linear relationships between variables, and robustly integrating this knowledge. The partial quantification of physicians' diagnostic and therapeutic experiences through AI has revolutionized the diagnosis and treatment of a wide array of diseases in recent years, sepsis being a salient example [75].

In 2021, Rainer König et al. [76] conducted a pivotal study on the IFNγ/IL10 ratio as a biomarker for categorizing septic shock patients in context to hydrocortisone therapy. Analyzing this ratio, they differentiated between patients benefiting from hydrocortisone and those not requiring it. A low IFNγ/IL10 ratio predicted increased survival in the hydrocortisone group, while a high ratio predicted better survival in the placebo group. Employing advanced machine learning methods and data from the CORTICUS trial [37], they confirmed the marker's validity in three clinical cohorts. The study established that using the IFNγ/IL10 ratio to guide hydrocortisone therapy notably improved 28-day survival rates. Additionally, it highlighted an inverse relationship between the IFNγ/IL10 ratio and pathogenic bacterial load, as shown through in vitro experiments and a mouse model. A high ratio correlated with lower bacterial load, indicative of the patient's immune status and infection severity. These findings offer a valuable guide for precision hydrocortisone use in sepsis management, potentially enhancing treatment efficacy and clinical outcomes.

In 2020, Pirracchio et al. [77] pioneered a study using machine learning to evaluate corticosteroids effectiveness in sepsis treatment. Analyzing data from five trials on glucocorticoid use in septic shock and RCTs comparing hydrocortisone with placebo (four RCTs were used to model and a fifth RCT was used to validate), they identified a machine learning-informed corticosteroid strategy that reduced absolute risk by about 2.9 %. The model demonstrated an AUC of 0.74 (95 % CI, 0.72–0.76) in the training set and 0.77 (95 % CI, 0.59–0.92) in the validation set, surpassing the Simplified Acute Physiology Score (SAPS II) models. A novel metric, NWT (any number willing to treat), was introduced, indicating improved outcomes when NWT exceeded 25. This approach effectively minimized glucocorticoid-related complications like hyperglycemia, hypernatremia, and metabolic alkalosis. However, the study's retrospective nature and reliance on data from four RCTs may limit generalizability. Nevertheless, the findings advocate for machine learning-driven, individualized glucocorticoid strategies in sepsis, signifying progress in personalized medicine.

In a meticulous study conducted by Stolarski et al. [78] utilized machine learning to forecast mortality risk in sepsis-affected animals. Post risk evaluation, subjects received either standard antibiotics and fluids (vehicle [VEH]) or a hydrocortisone, ascorbic acid, and thiamine combo (HAT). Notably, HAT didn't significantly lessen visceral dysfunction in a lung sepsis model, and varied sepsis types responded differently to it. Thus the cause of sepsis need be considered before initiating corticosteroid therapy. Using Lasso regression with 10-fold cross-validation, animals were classified into pDie and pLive groups based on vital signs at 6 and 24 h. The pDie group showed a 75 % death probability, contrasting with 35 % in the pLive group. Despite the lack of exact numerical details, the distinct mortality rate difference between groups indicated the model's effectiveness. However, the study's reliance on animal data limits its direct applicability to humans, necessitating further validation and controls for clinical relevance.

Wang et al. [79] introduced an innovative medical decision-making model that merges offline with deep reinforcement learning, overcoming the limitations of traditional reinforcement learning's interaction with dynamic environments. Utilizing the MIMIC-IV dataset, the model demonstrated a significant improvement in decision-making within a continuous state-action space. The authors evaluated survival rates through the utilization of the Q-function (a measure of the expected reward for taking a particular action in a given state). Their analysis indicated a Q-value of 52.47, aligning with a 0.844 survival rate, surpassing a clinician-assessed Q-value of 13.19 and 0.813 survival rate. The model's safety rate, assessed by AI-recommended drug doses' alignment with 70 % to 130 % of the clinician's strategy, was an impressive 0.902, underscoring its reliability. The study found the lowest mortality rate when clinician dosing matched the AI's recommendations. However, the retrospective nature of the study, based solely on the MIMIC-IV database, necessitates prospective studies for further validation and application of these significant findings.

In 2023, Bologheanu et al. [80] utilized a reinforcement learning algorithm to refine glucocorticoid protocols for septic shock in ICU patients, analyzing 3051 cases from the AmsterdamUMCdb database. The algorithm's recommendations showed a 59 % concordance with clinical treatments, advocating against corticosteroids in 62 % of cases, compared to clinicians’ 52 %. The algorithm further demonstrated its prowess by predicting a higher lower boundary of expected reward when juxtaposed with the clinician's decisions. Notably, the results extended into the test dataset, when patients' treatments aligned with the algorithm's recommendation, either to administer or refrain from corticosteroids, a marked reduction in mortality was observed. Specifically, concordance between the algorithm's decision and ICU physician's decision corresponded to an ICU mortality rate of 22.38 %, whereas discordance yielded a higher mortality rate of 28.33 %. This trend remained consistent regardless of whether corticosteroids were recommended (33.02 % vs. 34.27 %) or not (25.85 % vs. 32.22 %). The significance of this finding cannot be understated, adherence to the algorithm's guidance on corticosteroid use resulted in reduced mortality among ICU patients. This highlights the potential of tailored corticosteroid use in reducing ICU mortality, suggesting a shift towards precision medicine in sepsis treatment. However, the study's reliance on a single database, lack of external validation, and the need for broader generalizability assessment call for cautious interpretation. These findings underscore the promise of AI in healthcare, yet emphasize the necessity of further validation in clinical settings.

Kalimouttou et al. [81] utilized the LASSO model to evaluate the 2021 sepsis guidelines' recommendations, the aim of this study was to identify among the SCC guidelines the optimal bundle of recommendations that minimize 28-day mortality. Using data from the MIMIC-IV and eICU-CRD databases, the study compared the outcomes between the full treatment combination group (a bundle group that includes patients who were treated with at least all the recommendations selected in our bundle) (n = 2134) and the non-combination group (n = 2134). The full treatment group demonstrated a 28-day mortality rate of 15.5 %, significantly lower than the 37.8 % in the non-combination group, with an odds ratio (OR) of 0.41 [0.33–0.53] (p < 0.001). In the external validation cohort, the mortality rates were 11.3% for the full treatment group (n = 1680) and 15.1 % for the non-combination group, with an OR of 0.75 [0.60–0.94] (p = 0.02). The study concluded that adherence to the six recommended treatments—antimicrobials, balanced crystalloid, insulin therapy, corticosteroids, vasopressin, and sodium bicarbonate therapy—is linked to significantly lower mortality in sepsis patients, that is to say, the mortality rate of the bundle group receiving all six recommended treatments is lower than that of the no-bundle group, though further prospective validation is needed.

Table 3 summarizes multiple investigations concerning the utilization of AI in the glucocorticoid management of sepsis. The synthesized analysis of these studies manifests a promising efficacy in AI-augmented glucocorticoid therapy over traditional empirical approaches. Nevertheless, the intrinsic limitations across these studies warrant caution. These include the retrospective cohort design, reliance on a single-center open database, and other methodological constraints. Such shortcomings underline the necessity for more rigorous and comprehensive research. The practical application of these findings in real-world scenarios and in diverse databases remains embryonic and highlights a substantial chasm between the potential and current implementation.

**Table 3:** AI in glucocorticoid therapy for sepsis.

Title	First author	Objects and number of studies	Models	Corticosteroid regimen	Observations	Reference
Use of IFNγ/IL10 Ratio for Stratification of Hydrocortisone Therapy in Patients With Septic Shock	König	83 patients from the Berlin cohort, a subset of the CORTICUS study.	machine learning model, not specified	Patients received 200 mg/day hydrocortisone for 5 days followed by a tapering dose until day 11.	A low IFNγ/IL10 ratio indicated increased survival in the hydrocortisone group, whereas a high ratio suggested better survival in the placebo group.	[76]
Assessment of Machine Learning to Estimate the Individual Treatment Effect of Corticosteroids in Septic Shock	Pirracchio	2548 adult patients with severe infectious shock from 4 randomized controlled trials	Super Learner, with integrated machine learning algorithms.	1. IV hydrocortisone 50 mg/6 h for 5-7 days, tapered or not. 2. Same dosage plus oral flutriasone 50 mcg daily, no tapering.	Corticosteroids (hydrocortisone or hydrocortisone plus flutriasone) reduced 90-day death risk (RR 0.89, P = .004).	[77]
Machine learning and murine models explain failures of clinical sepsis trials	Stolarski	119 adult inbred wild-type ICR mice divided into two sepsis models: abdominal infection (CLP) and pneumonia (PNA)	Machine learning algorithms using Lasso regression and 10-fold cross-validation	Mice received hydrocortisone, vitamin C, and thiamine (HAT).	HAT benefited mice with abdominal infections but not those with pneumonia.	[78]
Learning Optimal Treatment Strategies for Sepsis Using Offline Reinforcement Learning in Continuous Space	Wang	6660 ICU patients meeting sepsis-3 criteria	Offline deep reinforcement learning based on continuous state-action space (including history capture models, generative models, perturbation models and Q-networks)	Use of hydrocortisone or not	Mortality was lowest when the dose matched the AI's decision.	[79]
Development of a Reinforcement Learning Algorithm to Optimize Corticosteroid Therapy in Critically Ill Patients with Sepsis	Bologheanu	3051 patients with sepsis admitted to the intensive care unit (ICU) were screened according to Sepsis-3 criteria	Reinforcement Learning Algorithms Based on Markov Decision Processes and Temporal Differences in Actor-Critic Methods	Systemic glucocorticoid doses were converted to hydrocortisone equivalents and categorized: 0 mg, 1-100 mg, 101-200 mg, 201-300 mg, and > 300 mg.	Mortality decreased when following the algorithm's steroid recommendations.	[80]
Machine-learning-derived sepsis bundle of care	Kalimouttou	42,735 adults with sepsis or septic shock	LASSO regression machine learning model	Antimicrobials, balanced crystalloid, insulin therapy, corticosteroids, pressor and sodium bicarbonate therapy	In the validation cohort and modeling cohort, the full treatment group had a lower 28-day mortality rate compared to the incomplete treatment group (15.5 % vs 37.8 % and 11.3 % vs 15.1 %).	[81]

### 3.4 Related databases and links

Contemporary investigations on the application of AI for enhancing glucocorticoid therapy in sepsis predominantly relies on publicly accessible datasets. This trend underscores the significance of open data in advancing scientific understanding in this domain. In alignment with this methodology, our team has integrated a variety of commonly used key datasets. This initiative is designed to streamline research endeavors, facilitating ease of access and in-depth exploration for future scholars in this crucial area of medical research. Our concerted efforts aim to foster a more cohesive and comprehensive understanding, potentially catalyzing breakthroughs in the effective management of sepsis through AI-augmented glucocorticoid therapy. The databases are shown in Table 4.

**Table 4:** Overview of relevant clinical medicine and bioinformatics databases.

Database name	Type	Brief description	URL
MIMIC-IV	Clinical medical data	Comprehensive clinical medical data covering over 40,000 patients. Includes but not limited to vital signs, laboratory test results, medication records, diagnostic information, and other clinical records.	https://mimic.mit.edu/
AmsterdamUMCdb	Clinical medical data	AmsterdamUMCdb is the first freely accessible European intensive care database. Contains data on 23,106 adult patients from 2003 to 2016, including demographics, vital signs, lab tests, and medications.	https://amsterdammedicaldatascience.nl/amsterdamumcdb/
eICU	Clinical medical data	eICU database gathers data from multiple intensive care units across the United States. Covers patients admitted to ICUs in 2014 and 2015. Includes data from over 200,000 patient admissions.	https://eicu-crd.mit.edu/
ArrayExpress	Bioinformatics data	A public database for storing and sharing functional genomics data, especially data derived from high-throughput gene expression experiments.	https://www.ebi.ac.uk/biostudies/arrayexpres
GEO	Bioinformatics data	A public database maintained by the National Center for Biotechnology Information (NCBI), for storing and freely sharing high-throughput gene expression data, including microarray and sequencing data.	https://www.ncbi.nlm.nih.gov/geo/
HiRID	Clinical medical data	HiRID is a free, publicly available dataset involving about 34,000 patient admissions to the ICU department of Bern University Hospital in Switzerland. Contains de-identified demographic information and a total of 681 routinely recorded physiological variables, diagnostic test results, and treatment parameters from January 2008 to June 2016.	https://hirid.intensivecare.ai/
PIC	Clinical medical data	PIC (Pediatric Intensive Care) database is a large single-center database containing admission information of patients in the ICU of Children's Hospital of Zhejiang University Medical College	https://github.com/Healthink/PIC

## 4 Future developments and challenges

### 4.1 Importance of glucocorticoids in the treatment of sepsis and existing problems

**Fig. 3:**
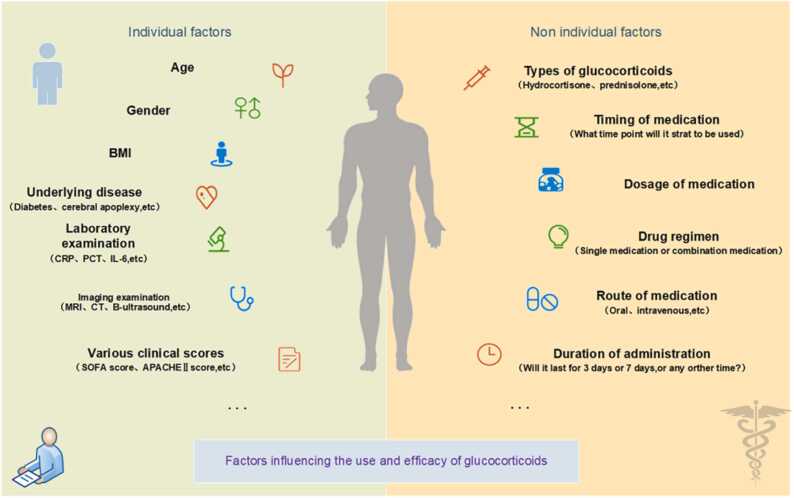
Glucocorticoid dosing and efficacy are governed by an intricate interplay of factors. As delineated in the figure, these span intrinsic patient-specific attributes and extrinsic determinants.

Various glucocorticosteroids and corresponding therapeutic regimens have long been deployed in the battle against sepsis. Kaukonen et al. [82] found in defining the SIRS criteria for severe sepsis that 1/8 of patients with infection-related organ dysfunction did not meet the SIRS criteria, indicating that not all patients with septic shock have an increased inflammatory response. Therefore, the steroid treatment for this group of patients should be considered separately. However, in this article, our study mainly focuses on sepsis patients who exhibit typical inflammatory responses. Despite decades of focused research and clinical exploration, the individual application of glucocorticosteroids remains shrouded in controversy, with numerous facets yet to be elucidated. When tailoring therapy to individual patients, questions persist regarding the optimal selection of the glucocorticoid type, the accurate determination of the dose, and the most effective mode of administration. This individualized approach necessitates further investigation, informed by both prior research findings and accumulated treatment experience, to foster a more nuanced understanding and enable the development of more precise, patient-centered interventions. Glucocorticoid dosing and efficacy is influenced by a number of factors, as shown in Fig. 3.

### 4.2 The potential of AI in optimizing glucocorticoid therapy for sepsis

The technological evolution of AI will contribute to the recognition of an increased number of disease subtypes, particularly in complex conditions like cancer and type 2 diabetes. With this diversification in disease classification. AI is anticipated to play a pivotal role in guiding the evolution of disease management towards more refined, tailored therapeutic interventions [83], [84]. This is particularly relevant in the context of sepsis, where the predictive accuracy and differentiation abilities of AI models frequently surpass traditional methodologies. The complexity and heterogeneity of sepsis manifest uniquely in different individuals due to factors such as the cause and degree of infection, representing a significant clinical challenge [85]. AI offers enhanced capabilities in navigating these intricate and diverse patterns.

Glucocorticoid therapy, a pivotal component in sepsis treatment, not only yields systemic effects [86] but also influences glucose metabolism and electrolyte imbalances [87]. The heterogeneity in treatment effects, often resulting from individual baseline characteristics, can cause considerable variation in therapeutic responses [88], [89]. This phenomenon is particular relevant in the administration of glucocorticoids for sepsis patients, where tailored approaches are ideal but conventional therapy is constrained by physician experience and prior authoritative trials [90], [91]. Group experiment results may not always translate to individual outcomes, with patient responses potentially determined by specific characteristics [92], [93]. This inconsistency introduces uncertainty, with different clinicians possibly prescribing disparate therapeutic strategies for the same patient.

AI presents a solution by generating decision-support models that can analyze a multitude of multidimensional factors. This process minimizes uncertainty stemming from empirical judgments and optimizes human resources by tailoring therapies to individual patients, thereby moving closer to the realization of personalized treatment [94]. It foresees a future where clinicians may predominantly supervise and audit AI-driven strategies.

However, this ideal scenario faces substantial challenges, necessitating further comprehensive research and innovation to fully materialize the potential of AI in individualized sepsis management.

### 4.3 Technical and ethical frontiers: navigating challenges in AI-driven healthcare

The aspiration for individualized treatment stands as a cardinal pursuit within medicine, a goal fraught with challenges given the multifactorial nature of disease and health. From genetic determinants to protein interactions, the roles of numerous factors in human physiology remain enigmatic [94]. The nascent stage of AI modeling research demands further development for real-world clinical translation. The interpretability of AI models, often likened to a black box that obscures the mechanisms translating inputs into outputs, has persisted as a critical concern [95]. Despite occasional demonstrating superiority over traditional models, this lack of transparency undermines the credibility of AI. Efforts to illuminate this black box are underway but necessitate continued exploration [96], [97].

Undoubtedly, AI in healthcare encounters distinct challenges, such as data privacy and security concerns. Common occurrences of data leaks and hacking underscore the importance of effective data protection strategies. Additionally, there's a risk of AI being used malevolently, exemplified by scenarios like manipulating AI to overdose diabetic patients with insulin, which is a critical area of concern [98]. Furthermore, the integration of AI models into clinical decision-making raises ethical considerations and relies heavily on extensive training data. Insufficient data can induce overfitting, leading to suboptimal performance in external test cohorts [99]. Navigating the complexities arising from AI-assisted decision-making requires comprehensive research, particularly in the demarcation of responsibilities and legal frameworks. Presently, legal provisions, scholarly literature, and information in this domain are scant, emphasizing the urgent need for further study and cultivation to realize the promise of AI in individualized medical care.

### 4.4 Future research directions for glucocorticoid therapy in sepsis: unexplored avenues and emerging issues

In accordance with the 2021 edition of the sepsis guidelines [56], adults experiencing infectious shock and necessitating continuous vasopressor treatment are advised to receive intravenous hydrocortisone at 200 mg per day, whether via segmented administration at 50 mg every six hours or continuous infusion. Despite this guidance, several pertinent questions warrant further exploration, including: (1) The optimal methodology for administering glucocorticoids - intravenous or continuous infusion - and the criteria for selecting the appropriate method for individual patients; (2) The determination of whether glucocorticosteroid dosages should be uniform for each patient; (3) An assessment of whether the types of glucocorticosteroids should remain consistent for every patient and the selection criteria thereof; (4) The establishment of parameters for determining the suitable duration of glucocorticosteroid treatment and stop time; (5) Role of mineralocorticoids; (6) Validation of a dose of 0.25 mcg/kg/min of vasopressor as the trigger for steroids. To address these queries, it appears paramount to devise real-time, personalized, and precise treatment strategies, considering each patient's unique characteristics and status. At present, the application of AI emerges as a likely tool to aid in decision-making within this domain. However, it must be acknowledged that AI modeling is in a nascent stage, lacking full maturity. Existing research bears inherent limitations, recognizing these constraints as indicative of the current study's limitations, future research must focus on overcoming these obstacles. Strategies may encompass incorporating additional prospective cohort studies to bolster the model's credibility, undertaking multi-center cohort investigations, and in relation to AI models, emphasizing exploration of their underlying mechanisms to enhance interpretability. Simultaneously, attention must be paid to delineating the ethical considerations associated with the deployment of AI.

### 4.5 Outlook for future research and applications

Upon examining the present state of AI research in the domain of glucocorticoid therapy for sepsis, it becomes evident that AI methodologies hold significant promise in delivering individualized and precise treatment. Such advancements stand to substantially reduce the uncertainty often encountered with traditional empirical approaches. However, caution must be exercised, as the majority of AI studies to date are retrospective, built upon existing databases, with some lacking external validation. These factors collectively constrain the credibility and broad applicability of AI models, presenting a formidable challenge in their validation and practical implementation within clinical environments [100].

AI harbors extensive potential within ICU sepsis management. However, the current phase of AI research in this field is not yet sufficient to support its routine clinical application, primarily due to lingering ethical considerations and issues concerning the uncertainty and interpretability of AI models. It underscores the imperative need for a concerted effort in clinical research, marked by an increase in prospective cohort studies, to ensure robust validation and comprehensive inclusion. Such efforts are vital to the continued evolution and responsible integration of AI within this critical healthcare sphere.

## Funding

This study was supported by 10.13039/501100001809National Natural Science Foundation of China (Grant No. 82272204), 5G Network-based Platform for Precision Emergency Medical Care in Regional Hospital Clusters funded by the 10.13039/501100006579Ministry of Industry and Information Technology of the People's Republic of China (2020NO.78), Key Clinical Specialty of Zhejiang Province in Critical Care Medicine (Grant No. Y2022), "Pioneer" and "Leading Goose" R&D Program of Zhejiang (Grant No. 2023C03084), 10.13039/501100005047Natural Science Foundation of Liaoning Province (Grant No. 2023-MS-288).

## CRediT authorship contribution statement

**Qi Zhao:** Conceptualization, Funding acquisition, Project administration, Supervision, Writing – review & editing. **Jingye Pan:** Conceptualization, Funding acquisition, Project administration, Supervision, Writing – review & editing. **Chenglong Liang:** Data curation, Investigation, Writing – original draft. **Shuo Pan:** Data curation, Investigation, Writing – original draft. **Wei Wu:** Data curation, Investigation, Writing – original draft. **Fanxuan Chen:** Data curation, Investigation, Writing – original draft. **Chengxi Zhang:** Investigation, Methodology, Visualization. **Chen Zhou:** Investigation, Methodology, Visualization. **Yifan Gao:** Investigation, Methodology, Visualization. **Xiangyuan Ruan:** Investigation, Methodology, Visualization. **Shichao Quan:** Conceptualization, Funding acquisition, Project administration, Supervision, Writing – review & editing.

## Declaration of Competing Interest

The authors declare that the research was conducted in the absence of any commercial or financial relationships that could be construed as a potential conflict of interest.
